# Monitoring Dynamic Recrystallisation in Bioresorbable Alloy Mg-1Zn-0.2Ca by Means of an In Situ Acoustic Emission Technique

**DOI:** 10.3390/ma15010328

**Published:** 2022-01-03

**Authors:** Dmitry Merson, Mikhail Linderov, Alexander Brilevsky, Alexey Danyuk, Alexei Vinogradov

**Affiliations:** 1 Institute of Advanced Technologies, Togliatti State University, 445020 Togliatti, Russia; D.Merson@tltsu.ru (D.M.); dartvi@gmail.com (M.L.); alexandrbril@yandex.ru (A.B.); alexey.danyuk@gmail.com (A.D.); 2 Department of Mechanical and Industrial Engineering, Norwegian University of Science and Technology, 4791 Trondheim, Norway

**Keywords:** magnesium alloys, high-temperature deformation, dynamic recrystallisation, acoustic emission, microstructure, mechanical properties

## Abstract

The tensile behaviour of the biocompatible alloy Mg-1Zn-0.2Ca (in wt.%) in the fine-grained state, obtained by severe plastic deformation via multiaxial isothermal forging, has been investigated in a wide range of temperatures (20 ÷ 300) °C and strain rates (5 × 10^−4^ ÷ 2 × 10^−2^) s^−1^ with the measurements of acoustic emission (AE). The dependences of mechanical properties, including the yield stress, ultimate strength, ductility, and the strain-hardening rate, on the test temperature and strain rate, were obtained and discussed. It is shown for the first time that an acoustic emission method is an effective tool for in situ monitoring of the dynamic recrystallisation (DRX) process. The specific behaviour of the acoustic emission spectral density reflected by its median frequency as a function of strain at various temperatures can serve as an indicator of the DRX process’s completeness.

## 1. Introduction

Due to their exceptional properties’ profile, magnesium alloys attract burgeoning attention for various applications that span from light vehicle fuel-saving transportation (aerospace, automotive, high-speed rail transport, etc.) to consumable electronics and bio-medical devices. The excellent biocompatibility of magnesium and its ability to gradually dissolve in biological media without adverse effects on human tissues opens a potentially massive market for temporary medical structures, such as orthopaedic implants and cardiovascular stents. However, the requirements for biomedical materials are particularly stringent because, besides biocompatibility, non-toxicity, controlled bio-degradability, etc., these materials are supposed to hold the integrity of implanted structures over the whole period of healing, i.e., while providing the necessary mechanical support to healing tissues, they must possess reasonably high resistance to static and dynamic loads in body fluids. These requirements imply that the materials aimed at biomedical applications are supposed to have:

(1) High purity, assuming the absence of toxic or potentially harmful elements that should not enter the human body during the resorption of the implanted structure [1,2].
(2) Sufficiently high strength and ductility, which are conventionally supposed to be better than 250 MPa and 15%, respectively [3]. High ductility is necessary to achieve the desired products’ shape during manufacturing, which traditionally involves metal forming operations. In addition, remaining ductile is a prime requirement for the final product as shape correction and adjustment may be needed during location-specific deployment.
(3) The controllable (tuneable) rate of bio-corrosion (resorption), which is pivotal for the medical application of magnesium alloys as temporary structures. This rate should be neither too slow nor too fast and comply with the rate of tissue regeneration. Hence, it is commonly assumed to be not worse than 0.5 mm/year in vivo [3].

Significant efforts invested into the research on biodegradable Mg alloys are reflected in the growing number of publications (see recent comprehensive reviews [4,5,6,7,8] and references therein covering the state-of-the-art and highlighting the existing challenges within the area) [9]. Despite all challenges, it is safe to state that the basic principles of creating bioresorbable magnesium materials with the necessary combination of functional properties have been formulated, and a wide variety of biocompatible and bioresorbable alloying systems have been proposed in the literature. Of many existing variants, the design strategy of lightly alloyed Mg-Zn-Ca proposed by Uggowitzer and co-authors in a series of publications [10,11,12,13,14,15] is to be mentioned as one of the most viable and effective concepts. Zinc has long been recognised as one of the most effective alloying elements in Mg-based alloy compositions. With its relatively high maximum solubility in magnesium (of 6.2 wt.%), zinc is undeniably an important solution strengthening agent increasing the critical resolved shear stress (CRSS) for basal slip. However, its function in Mg alloys is much broader than just providing a strengthening effect. For example, zinc is beneficial in overcoming the harmful effect of Fe and Ni impurities, thus increasing the overall corrosion resistance [16,17]. Calcium is the main component of human bone. It can strengthen the Mg matrix by combining solution strengthening, grain boundary strengthening and precipitation strengthening, though only moderately. Due to its low density (1.55 g/cm^3^), Ca imbues the Mg-Ca alloy system with the advantage of having a similar density to the bone. However, the prime function of Ca in the tertiary Mg-Zn-Ca systems lies in the formation of the specific intermetallic compounds affecting virtually all physical and (bio) chemical properties of the alloys [12,13,14,15]. Furthermore, the alloys with high Zn content suffer from rapid biocorrosion due to the abundant presence of the intermetallic phase Mg_6_Zn_3_Ca_2_. With the Zn content reduced to 1 wt.% or less, the (Mg,Zn)_2_Ca phase prevails over Mg_6_Zn_3_Ca_2_. As it is less noble than the Mg-matrix, this phase does not act as a cathodic site, in contrast to Mg_6_Zn_3_Ca_2_. Homogeneously distributed Ca-containing precipitates influence dynamic recrystallisation and grain growth during hot processing, leading to the favourable formation of uniform microstructures. Finally, lean alloys with an attractive combination of high strength, ductility and superior bio-corrosion resistance have been produced, taking advantage of the combined effect of solid-solution hardening, grain-boundary hardening, and precipitation hardening [15].

Deformation processing is an essential component of the fabrication of any wrought alloy. However, it becomes of particular significance for the low-alloyed compositions, e.g., when the small concentration of Zn (of 1 wt.%) and Ca (<0.3%) is used to achieve the highest corrosion resistance. While conventional direct extrusion is by far the most popular process employed for the fabrication of Mg alloys, the unbeatable combination of high mechanical performance and corrosion resistance can be achieved by using a variety of severe plastic deformation (SPD) techniques [18], which are readily available for practitioners. These techniques have been used in different combinations to achieve extreme microstructure refinement, form a favourable crystallographic texture and homogenise the distribution of second phase particles contributing to the strength and controlling the overall corrosion resistance [19,20,21,22].

The development of perspective alloys is just the first task in the long chain of manufacturing operations, including a comprehensive characterisation of the microstructure and properties as necessary steps before the final medical devices can be produced and brought to market. Depending on the application, the implanting structures vary broadly in shapes and dimensions, which can often be as small as a few millimetres or sub-millimetres in one or two directions (e.g., wires, tubes, foils, scaffolds, etc.). The fabrication of products with such small dimensions involves metal forming through drawing, rolling or other processes to giant strains, assuming significant workability of the alloy. The ductility-permitting plastic deformation to large strains is often achieved at particular (usually narrow) temperature–strain rate regimes, which are not known a priory and need to be determined experimentally. Even though the significance of dynamic recrystallisation (DRX) has long been recognised for the mechanical behaviour and microstructure formation in Mg alloys at elevated temperatures [23,24,25], until recently, the number of reports documenting the mechanical response of perspective magnesium alloys in a wide temperature–strain rate range is still scarce. For example, some specific SPD schemes have been explored to find the optimised processing conditions [26,27]. Several recently emerged papers where the temperature–strain rate behaviour of magnesium alloys has been studied in considerable detail are to be mentioned. Ding et al. [28] established the optimal parameters of hot work deformation of the extruded AZ31 magnesium alloy in uniaxial compression in the temperature range of 250 to 500 °C at strain rates of 0.005–5 s^−1^. Backed by microstructural observations, the following guiding regimes have been formulated for the processing of AZ31: the temperature range is 300 to 400 °C, and the strain rate range is 0.005 to 0.05 s^−1^. Zhang et al. [29] studied the thermomechanical behaviour of AZ31B and Elektron 717 Mg-Zn-Re-Zr alloys under hot forging conditions. These authors showed that dynamic recrystallisation accompanied by notable texture evolution occurred during deformation at a high homologous temperature in both alloys. These two interrelated processes were identified as key factors influencing the viscoplastic behaviour where the yield stress of both studied alloys decreased with increasing temperature and decreasing strain rate, while the ductility of the samples increased concurrently.

Aliyari et al. [30] investigated the behaviour of the potentially biocompatible alloy Mg-0.35Y-2.17Nd-0.36Zr (in wt.%), which was hot extruded and then tested in tension in the temperature range of 225 to 525 °C and strain rates which varied between 3 × 10^−4^ and 3 × 10^−2^ s^−1^. The deformation mode altered from the predominant twinning to dislocation slip when the temperature increased above 325 °C. Kaviani et al. [31] investigated the effect of hot deformation parameters (temperature and deformation rate) on the mechanical and corrosion properties of the biocompatible Mg-4Zn-0.5Ca-0.75Mn alloy. They showed that an increase in the deformation temperature positively affects corrosion properties. Chaman-Ara et al. [32], using the extruded Mg-Zn alloy with Y additions, investigated its microstructure and mechanical properties during hot compression in the temperature range of 300 to 450 °C and strain rates of 0.001 to 1 s^−1^. The deformation maps were constructed with optimal deformation conditions highlighted for enhanced deformability. The addition of Y reduced the stress for the onset of DRX. Axial compression testing was used in the above-cited papers. Although it was noted in [32] that the deformation mode (compression or tension) does not significantly affect the processing maps for hot deformation of the alloy AZ31, the same assertion cannot be immediately generalised and expanded to the entire class of Mg alloys due to a well-known strong asymmetry in their mechanical response in tension and compression [33].

The complexity of the deformation behaviour of magnesium alloys is associated with the low symmetry of the hcp lattice resulting in the plurality of active deformation mechanisms involved in different combinations at different deformation stages [34]. Dislocation slip 〈a〉 on the most densely packed basal plane (0001) in the most closely packed direction is the dominant deformation mode at low homologous temperatures [35,36]. However, basal slip provides only two independent slip systems, which are not enough to accommodate the imposed plastic strain homogeneously. Even when all basal and non-basal systems are activated, the maximum four independent slip systems are activated [37,38], which is still insufficient to accommodate arbitrary homogeneous deformation of a polycrystalline aggregate according to the Taylor principle, which requires at least five independent slip systems [39]. Hence, non-basal slip systems, i.e., pyramidal slip and/or mechanical twinning, which is possible in several independent variants, must be activated. All these mechanisms have different critical resolved shear stresses and respond differently to testing temperature and strain rate. The complexity of the multi-faceted behaviour and mutual interplay between different deformation modes is far from being understood. The picture becomes particularly complicated when dynamic recrystallisation comes onto the stage, modifying the distribution and shapes of grains, their crystallographic orientation and the deformation mechanisms involved.

Investigation of the relationship between the microstructure resultant from the thermo–mechanical processing of an Mg-alloy and the deformation mechanisms occurring together with dynamic recovery and dynamic recrystallisation is a laborious task. To generate a comprehensive picture, one needs to obtain a large array of microscopic data with many combinations of test parameters: loading rate, temperature, imposed strain, etc. This work can be significantly facilitated if the microscopic studies are carried out in a guided way, that is, when critical conditions corresponding to the onsets of qualitative changes in deformation processes or the microstructure are indicated through independent in situ measurements. In order to determine such breakpoints, the method of acoustic emission (AE) is well suited since this AE phenomenon is intimately related to the local dynamic stress relaxation processes occurring during microstructural rearrangements in the material [40,41,42]. In the present work, we employ this method (for the first time, to the best of our knowledge) to investigate the deformation behaviour of the Mg-Zn-Ca alloy at elevated temperatures in situ.

## 2. Materials and Methods

### 2.1. Alloy Processing

A popular Mg-Zn-Ca alloying system [13,43,44] was chosen for the present study. After appropriate thermomechanical treatment, alloys of this kind have an excellent balance between mechanical and corrosion properties [45,46,47,48,49]. The alloy was cast from commercially pure components, and the chemical composition of the alloy-Mg-0.85Zn-0.15Ca-0.012 Zr (all in wt.%) was determined by the optical emission spectrometer ARL 4460 OES (ThermoFisher Scientific, Waltham, MA, USA). Zirconium was added to the melt to reduce the content of iron to be below the tolerance limit [50,51]. In addition, Zr is known as an efficient grain refining agent. The amount of all trace elements did not exceed 0.1 wt.%.

**Figure 1:**
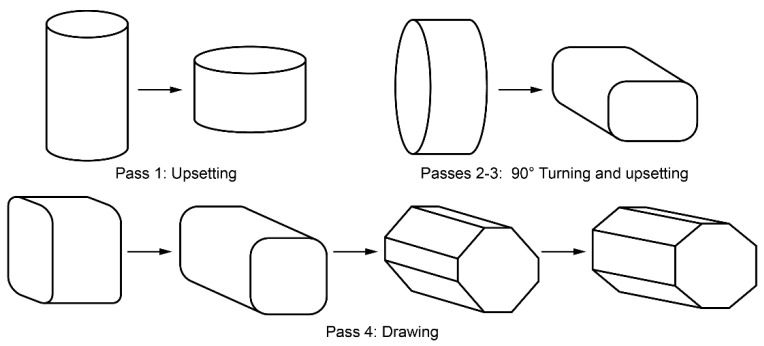
Schematics of the multi-axial forging cycle.

The as-cast alloy was homogenised at 430 °C for 12 h in argon. The billets of 150 mm length and 170 mm diameter were subjected to multiaxial isothermal forging (MIF) to 20 cycles with the total equivalent strain per cycle of 1.4 [52,53]. Each cycle comprised four forming operations, illustrated in Figure 1. The billet was preheated to 425 °C and the first four MIF cycles were carried out at this temperature. The following cycles were performed with a 25 °C temperature reduction after every four cycles with the final cycles carried out at 325 °C [54]; finally, the billet was upset at 325 °C and cut into plates.

### 2.2. Mechanical Testing

**Figure 2:**
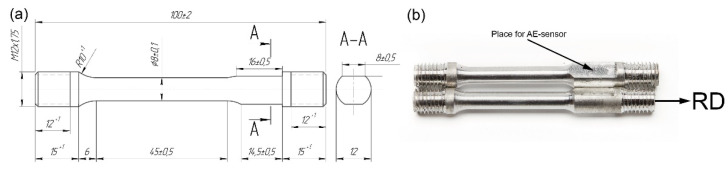
Schematic drawing (**a**) and photographic images (**b**) of the samples.

The standard tensile threaded I-shaped specimens with a round cross-section (as per recommendations of the ASTM E8/E8M-21 standard) were machined from the plates, as shown in Figure 2. The longitudinal axis of the specimens corresponded to the direction of the last upset operation (denoted as RD in analogy with the rolling direction). A flat facet of 16 mm length was machined on the shoulder part of the specimen to mount the AE sensor in proximity to the gauge part.

**Figure 3:**
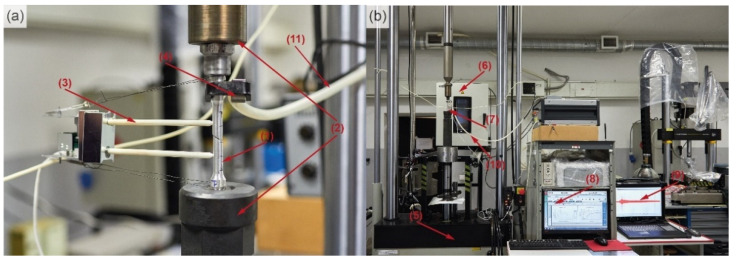
Experimental setup: (**a**) magnified view of the specimen and (**b**) general view of the testing facilities; (1)—specimen; (2)—high-temperature grips; (3)—extensometer with ceramics probes; (4)—AE sensor; (5)—Instron 8802 servohydraulic frame; (6)—environmental chamber; (7)—thermocouple; (8)—test-controlling PC; (9)—AE system; (10)—AE sensor clamp; (11)—air-chilled thermal insulating shell for the AE signal cable.

The experimental setup is shown in Figure 3 with key components are numbered and described in the caption. The specimens (1) for the uniaxial tensile tests were mounted in the heat-resistant grips (2) on a PC-controlled servohydraulic universal tensile machine Instron 8802 (Instron, Norwood, MA, USA) (5) equipped with an environmental chamber Instron 3119-406 (Instron, Norwood, MA, USA) (6) and BlueHill 3.0 software (Instron, Norwood, MA, USA). The strain was measured by a clip-on high-temperature extensometer Epsilon 3448 (Epsilon, Jackson, MO, USA) (3). Tests were performed under a crosshead velocity-controlled mode at nominal strain rates of 5 × 10^−4^, 5 × 10^−3^ and 2 × 10^−2^ s^−1^, and temperatures 20, 100, 150, 200 and 250 °C. One specimen was tested at 100 °C at 2 × 10^−2^ s^−1^ to confirm the general trends. Two thermocouples (7) were attached to both sides of the specimen to ensure temperature stability to be better than ±2 °C.

### 2.3. Acoustic Emission Method

For AE measurements, the piezoelectric sensor MSEA-1300WB (Microsensors AE, Sarov, Russia), Figure 3a(4), calibrated as described in [55], was attached to the shoulder part of the specimen (1) with the elastic clamp (10). Vacuum oil was used as a coupling medium to ensure the efficient transfer of elastic waves from the surface to the transducer. The AE signals were amplified by 60 dB in the frequency band from 10 to 1200 kHz by the low-noise pre-amplifier 2/4/6 and recorded by the 16-bit PCI-2 data acquisition board (MISTRAS, Princeton, NJ, USA), Figure 3b(9), operating in the “thresholdless” mode at the sampling rate of 2 Msamples/s in the frequency band of 100–1000 kHz. An additional +6 dB gain was set on board. The AE measurements at elevated temperatures are challenging as virtually all commercially available so-called high-temperature sensors have a significantly compromised sensitivity and/or bandwidth. The LiNb-based piezoelectric element utilised in the sensor MSAE-1300WB with a frequency band of 50–1300 kHz is featured by its high Curie temperature and sensitivity comparable to that of most commercial PZT transducers [55]. Furthermore, the coaxial cable has a thermal insulating shell, which was air-cooled during testing, Figure 3a(11). The cable temperature was measured by a thermocouple attached to the hot side of the shall. The pressure and the rate of the cooling air were kept constant during the test.

The continuously streamed data were sectioned into consecutive individual realisations of 4k samples without overlapping. A Fourier power spectral density (PSD) function G(f) was then calculated from these data using a Welch method. The average per realisation *AE* energy *E* was calculated from the corresponding PSD as $E_{AE}=T\int_{f_{\min}}^{f_{\max}}G(f)\;df$ (*T* is the length of the realisation). The median AE frequency $f_{m}$ was introduced according to the definition $\int_{0}^{f_{m}}G(f)\;df=\int_{f_{m}}^{\infty }G(f)\;df$, see [56] for details. Both E and $f_{m}$ values were obtained from G(f) after subtraction of the PSD of the laboratory noise pre-recorded before the start of loading during each test.

### 2.4. Microstructure Characterisation

The X-ray diffraction (XRD) technique was employed for the phase analysis using the Shimadzu Maxima XRD-7000 (Shimadzu, Kyoto, Japan) diffractometer with a goniometer in the Bragg–Brentano geometry with a secondary curved graphite monochromator (long focus tube with CuKα radiation, 40 mA current and 40 kV accelerating voltage, respectively). Scanning was carried out over the 10–90° 2θ range at 0.5 deg/min scan speed with 0.02 deg steps. The phases were identified using the Shimadzu PDF2 database.

The sections for the EBSD analysis were cut from the middle of the gauge part of the tensile specimens normally to the longitudinal axis (RD in Figure 2). All sections were gently mechanically ground by SiC paper down to #2500-grade and then further polished with diamond pastes down to 0.25 μm particle size to a mirror-like finish. Finally, the surface of the sections was ion milled by a Hitachi IM4000 Plus system (Hitachi, Tokyo, Japan) using a low-energy Ar+ ion beam. The optimum milling conditions were found with the 3° inclination angle to the surface, 6 kV acceleration voltage and 1.5 kV discharge voltage, 0.1 cm^3^ min^−1^ argon gas flow and 25 rpm for 1–2 h milling time.

The microstructure in the initial state after straining at elevated temperature was investigated by the field emission gun scanning electron microscope (SEM) SIGMA (Zeiss, Jena, Germany) equipped with the Hikari electron backscattering diffraction (EBSD) camera (EDAX/TSL, Mahwah, NJ, USA) and orientation image microscopy software package OIM-6.2 from the same company. The EBSD maps were obtained from the regions of 160 × 160 μm^2^ with a step size of 250–350 nm. The indexed points with a confidence index less than 0.1 were ignored in post-processing. The grains were identified using a minimum misorientation angle of 5°, and the grain diameter was determined from the grain area, assuming a spherical grain. The grain boundaries were defined by means of misorientation between the adjacent points, with boundaries having a misorientation between 2°–15° regarded as low angle grain boundaries (LAGBs) and boundaries with a misorientation larger than 15° evaluated as high angle grain boundaries (HAGBs). For the grain identification procedure, the criterion for reliability was used with not less than 6–8 points with co-directional orientation within 5 degrees of misorientation [57].

## 3. Results

**Figure 4:**
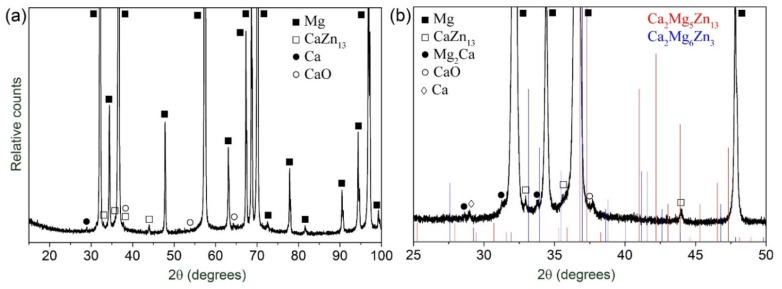
The X-ray diffraction pattern of the as-fabricated Mg-1Zn-0.2Ca alloy: (**a**) full scan, (**b**) fragment of the XRD pattern obtained at a slower scan speed of 0.1 deg/min with a scan step of 0.005 deg.

The result of the XRD scan shown in Figure 4 revealed distinct peaks corresponding to α-Mg. The reflexes of CaO oxide and the CaZn_13_ phase were found in the XRD pattern. However, in good agreement with thermodynamic predictions [15], there was no sign of the tertiary intermetallic compounds, such as Ca_2_Mg_6_Zn_3_ phase or Ca_2_Mg_5_Zn1_3_, Figure 4b, which are commonly seen in many Mg-Zn-Ca alloys with a higher content of Zn and Ca [48,58,59]. However, traces of a small amount of the Mg_2_Ca phase and undissolved Ca particles were found in the diffractograms, which were particularly well visible on the diffraction pattern obtained with a higher resolution, Figure 4b.

**Figure 5:**
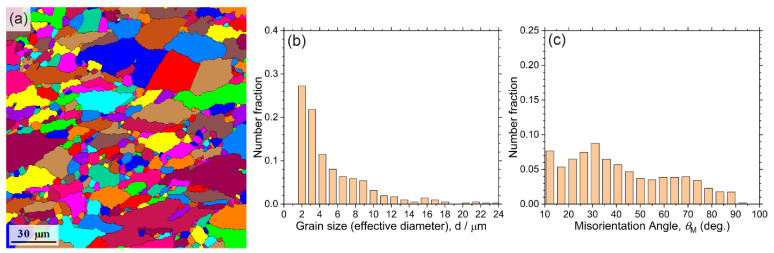
Initial microstructure of the Mg-1Zn-0.2Ca alloy (**a**) and its parameters: (**b**)—grain size distribution; (**c**)—distribution of the angles of misorientation between grains.

The initial microstructure was characterised by a wide distribution of grain shapes and dimensions, Figure 5a. Although the grains size distribution was unimodal, the effective grain diameters varied from 1 to 25–30 μm, Figure 5b. The maximum of the histogram lies in the fine grain domain corresponding to grain sizes of 2–3 μm. The misorientation angles were distributed nearly evenly, with the broad peak around 30°. This microstructure indicated that the recrystallisation process during isothermal forging operations had not been completed. The large grains appeared elongated in the plane of the last upsetting (horizontal direction in Figure 5c).

### 3.1. Mechanical Behaviour at Ambient and Elevated Temperatures

**Figure 6:**
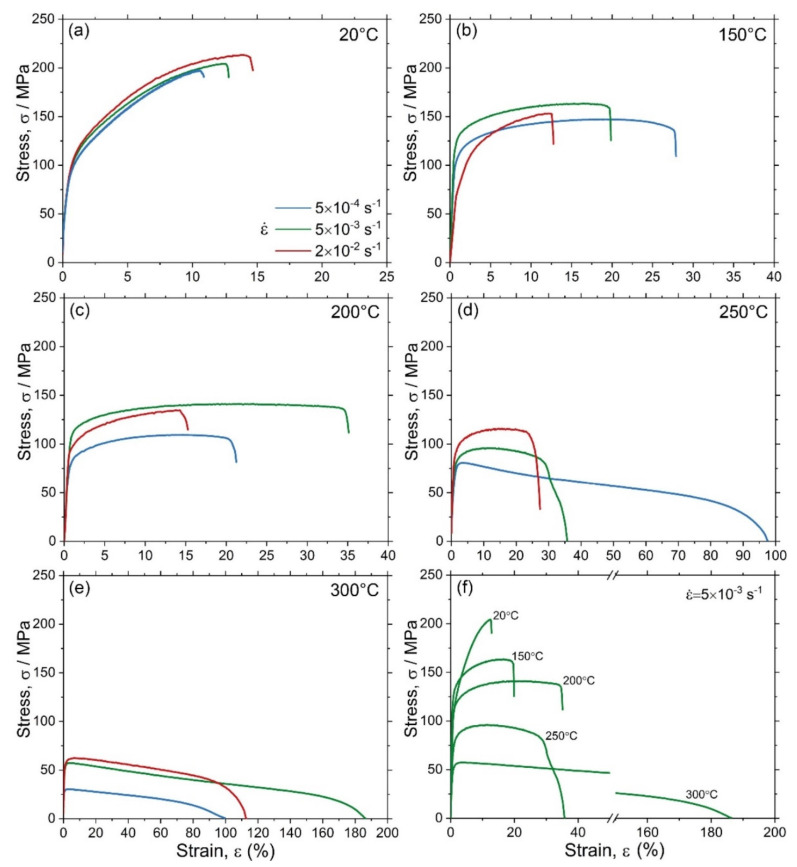
Tensile diagrams for the specimens tested at various temperatures and three strain rates (the colour coded legend shown in (**a**) applies to all plots): (**a**) 20 °C, (**b**) 150 °C, (**c**) 200 °C, (**d**) 250 °C, (**e**) 300 °C, and (**f**) the summary plot showing the evolution of the σ(ε) curves obtained at $\dot{\varepsilon }$ = 5 × 10^−3^ s^−1^ with increasing test temperature.

Engineering stress–strain diagrams for uniaxial tension at various temperatures and strain rates are summarised in Figure 6a–e. The conventional yield stress $\sigma_{0.2}$, ultimate tensile strength $\sigma_{UTS}$, elongation at break $\varepsilon_{f}$ and the average strain-hardening rate $\theta_{3-10}$ determined between 3% and 10% strain are represented in Table 1. To ease the observation of changes in the tensile diagrams with temperature, Figure 6f shows the σ(ε) curves obtained at the same strain rate of 5 × 10^−3^ s^−1^ and different temperatures. As expected, the tensile strength decreased, while the elongation to failure increased with increasing temperature. Most notable changes in the mechanical response were observed starting from 250 °C. The interesting result is that the yield stress increased at 150 °C compared to that at ambient temperature and then decreased with increasing test temperature. This anomaly in the behaviour of the yield stress has yet to be understood on the basis of detailed microstructural examinations, which are beyond the scope of the present work, and which will be the focus of our further investigations.

### 3.2. Acoustic Emission

**Figure 7:**
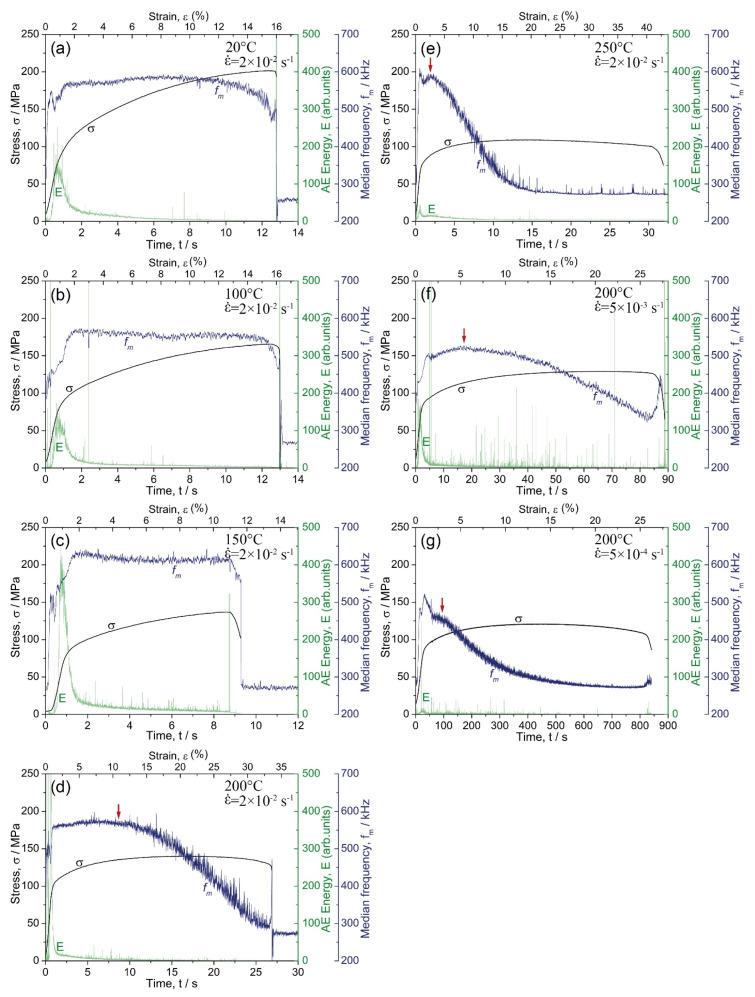
The AE energy and median frequency behaviour as a function of deformation time (strain) in tension at 2 × 10^−2^ s^−1^ strain rate and testing temperatures 20 (**a**), 100 (**b**), 150 (**c**), 200 (**d**) and 250 °C (**e**); plots (**f**) and (**g**) show the same parameters for testing at 200 °C and $\dot{\varepsilon }$ = 5 × 10^−3^ s^−1^ and 5 × 10^−4^ s^−1^, respectively. Arrows in (**d**–**g**) indicate the breakpoints in the $f_{m}$ trends from ascending to descending at the strain $\varepsilon_{cr}^{AE}$.

Figure 7 shows the typical behaviour of the AE spectral characteristics represented by the energy and median frequency in dependence on time (or strain) synchronised with the respective σ(ε) diagrams. To get a better understanding of the effects of temperature and strain rate on AE, Figure 7a–e compare the results obtained for the same strain rate of 2 × 10^−2^ s^−1^, but different temperatures. Moreover, Figure 7d,f,g illustrate the data obtained with the strain rates varying at the same test temperature of 200 °C. For the tests performed at 250 °C at the lowest strain rate (as well as for all tests performed at 300 °C), the AE level was quite low, i.e., close to the background noise. Therefore, these results were excluded from the analysis in the present work.

**Figure 8:**
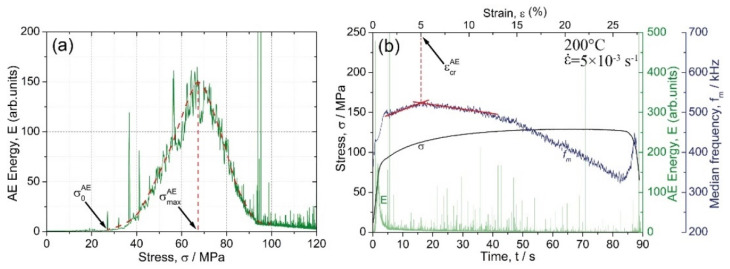
Schematic of determining the AE characteristics using the example of testing results obtained at 200 °C and $\dot{\varepsilon }$ = 5 × 10^−3^ s^−1^; (**a**) stress corresponding to the onset of AE $\sigma_{0}^{AE}$ (AE elastic limit), and stress corresponding to the AE peak $\sigma_{\max}^{AE}$ (AE yield stress); (**b**) critical strain $\varepsilon_{cr}^{AE}$ corresponding to the breakpoint in the $f_{m}$ behaviour.

Typically, for most metallic materials [60], during loading of the alloy Mg-1Zn-0.2Ca, the AE energy peaked shortly after the onset of plastic yielding and decayed gradually afterwards in response to the growing dislocation density and a concomitant decrease in the dislocation mean free path. Concurrently, the AE spectrum shifted to a higher frequency domain, as was reflected by the steady increase in the median frequency with strain up to the onset of necking instability. Figure 8a illustrates the formation of the AE peak as a function of the flow stress σ. A test carried out at 200 °C and 5 × 10^−3^ s^−1^ strain rate was used as a representative example. One can see that the AE peak has a nearly symmetrical bell shape in the E−σ coordinates. Two characteristic AE features are worthy of notice: the stress corresponding to the first departure of the AE signal from the noise level $\sigma_{0}^{AE}$, and the stress corresponding to the AE maximum $\sigma_{\max}^{AE}$. Ideally, these two quantities define the lowest detectable stress when plastic deformation activates in local volumes of the material (notably, far below the conventional yield stress), and the stress when the entire undeformed volume becomes involved in plastic yielding. Commencing locally in individual grains, which were most suitably oriented for dislocation initiation or contain residual stress concentrators, plastic flow expanded to the entire volume with increasing stress. These two specifically determined stresses can be referred to as the AE elastic limit and the AE yield stress, respectively.

As reflected by the AE median frequency, the AE spectral density exhibited a clear shift to a high-frequency domain in parallel with the flow stress as long as plastic deformation was mediated by dislocation slip during the uniform deformation stage [61,62,63]. This trend broke in three situations: (i) when the mechanisms other than dislocation slip came onto the stage, e.g., twinning [34,64,65], or martensitic transformation in TWIP and TRIP steels [66,67] (it has been shown in the cited works that when profuse twinning is observed, the $f_{m}$ value tends to level out); (ii) when the strain localisation occurred, and the plastic flow was governed by substantially correlated dislocation motion [68], and (iii) if, for any reason, the dislocation mean free path increased as opposed to the strain hardening process. One can see that the AE median frequency behaved notably differently with straining at different testing temperatures. At ambient and relatively low temperatures of 100 and 150 °C, the AE median frequency increased at the onset of plastic flow and tended to saturate with only a slight (or no) trend to decrease at the mature deformation stage (a similar finding has been reported formerly for the alloy ZK60 deformed at room temperature [61,62]). However, at a temperature equal to or higher than 200 °C, the behaviour of the median frequency changed drastically from the steady flow to the rapid decrease after a certain critical strain $\varepsilon_{cr}^{AE}$, Figure 7d,e. Moreover, the appreciable decrease in $f_{m}$ commenced at lower strains as the strain rate decreased, Figure 7g,f. Figure 8b illustrates the scheme of how $\varepsilon_{cr}^{AE}$ was determined. Values of $\sigma_{0}^{AE}$, $\sigma_{\max}^{AE}$ and $\varepsilon_{cr}^{AE}$ determined from AE measurements are shown in Table 1 together with the corresponding data derived from the stress–strain curves under different testing conditions.

## 4. Discussion

**Figure 9:**
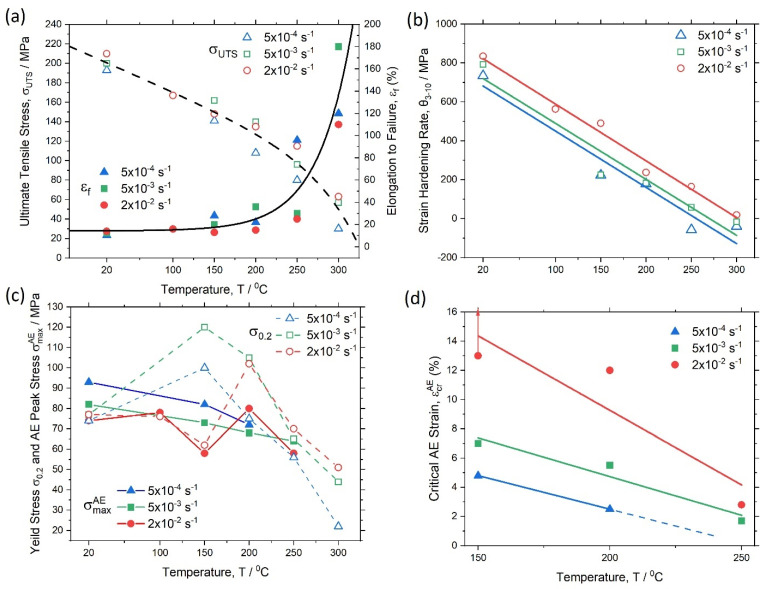
Dependence of mechanical properties and AE features on the test temperature: (**a**) ultimate tensile strength $\sigma_{UTS}$ and elongation at break $\varepsilon_{f}$, (**b**) average strain hardening rate *θ*_3–10_, (**c**) conventional yield stress $\sigma_{0.2}^{}$ and AE (or physical) yield stress $\sigma_{\max}^{AE}$, and (**d**) critical strain $\varepsilon_{cr}^{AE}$.

As has been mentioned, the alloy strength decreased with increasing test temperature while the ductility increased simultaneously. The change in these quantities appeared to be most pronounced at 250 °C and higher temperatures, Figure 9a. Trends were the opposite with an increasing strain rate: strength increased while ductility decreased, as is commonly seen for structural materials where deformation is mediated by thermally activated mechanisms, such as dislocation slip. The average strain hardening rate decreased nearly linearly with increasing temperature for all strain rates used in the tests, Figure 9b. However, the effect of strain rate was more pronounced at higher strain rates.

While many mechanical properties exhibited stable trends with increasing test temperature ($\sigma_{UTS}$,$\varepsilon_{f}$, *θ*_3–10_), the properties demonstrating the most irregular behaviour were those reflecting the elastic–plastic transition: the conventional yield stress $\sigma_{0.2}^{}$ and the “physical” yield stress corresponding to the peak AE $\sigma_{\max}^{AE}$, Figure 9c. One can see that these characteristics behaved most irregularly at 150 °C, i.e., their values sporadically and sharply varied for different strain rates. The stress corresponding to the appearance of a stably detectable AE signal (elastic limit related likely to the CRSS in the most favourably oriented grains) showed an even greater scatter. It is highly likely that this irregular behaviour of stress-related characteristics at the very early deformation stage is associated with the competition between two alternative deformation modes—basal slip and twinning [69,70,71,72,73,74]—having comparable yet slightly different CRSS depending on temperature and strain rate (admittedly, finding the CRSS values precisely in polycrystals is a difficult task [75]). While basal slip was the predominant deformation mechanism in Mg, profuse twinning activated at low homologous temperatures, whereas a combination of basal and non-basal slip modes governed the plastic flow at high temperatures. One can suppose that the competition between sip and twinning becomes most fierce at 150 °C for a given alloy. Jain and Agnew [76] and Chapuis and Liu [77] showed that for the alloy AZ31, the CRSS value for $\left\{10\bar{1}2\right\}$ tension twinning increased with temperature, and $\left\{10\bar{1}1\right\}$ compression twinning did not show up above 150 °C. This finding, although it is promising and nicely illustrating the capacity of the modern AE technique to detect small scale plastic events at the very early deformation stage, and, thereby, to clarify the CRSS value for the corresponding mechanisms, addressing this issue is beyond the scope of the present work.

Figure 9d shows the dependence of the critical strain $\varepsilon_{cr}^{AE}$ (corresponding to the beginning of the decrease in $f_{m}$) on the test temperature (notice that at 150 °C, such a point was not found; therefore, the elongation at break is shown in Figure 9d for this temperature). As can be seen, the $\varepsilon_{cr}^{AE}$ value persistently decreased with an increase in temperature and/or decrease in strain rate. As has been discussed above, the AE median frequency is a spectral feature characterising the relaxation time in the 1st order stochastic autoregressive process of AE generation [61], which is governed by the inter-obstacle spacing, i.e., by the mean free path of dislocations. Therefore, as has been discussed above, the $f_{m}$ value tends to increase monotonically in response to the increase in the accumulated dislocation density as strain hardening proceeds. The present observations, however, showed the break in this common trend: starting from the critical strain $\varepsilon_{cr}^{AE}$, $f_{m}$ decreased, indicating unequivocally that the mean free path of dislocations increased. Perhaps, the only rational explanation for this behaviour would assume that partial recovery occurs in the microstructure as a result of dynamic recrystallisation. If this is the case, then the strain $\varepsilon_{cr}^{AE}$ could be related to the coarsening of recrystallised grains, which, in the just recrystallised state, were free from dislocations.

To verify this hypothesis, the following additional experiments were performed. For each of the three test temperatures (150, 200 and 250 °C), three interrupted tensile tests were carried out. The tests were terminated when the following conditions on strains were met at the critical $\varepsilon_{cr}^{AE}$ for a given strain rate, as well as at the lower and higher strains denoted in Table 2 as $<\varepsilon_{cr}^{AE}$ and $>\varepsilon_{cr}^{AE}$, respectively. After unloading, the sections were cut from the middle of the gauge part of the specimens as described in Section 3.1, and the microstructure was investigated by the EBSD technique.

**Figure 10:**
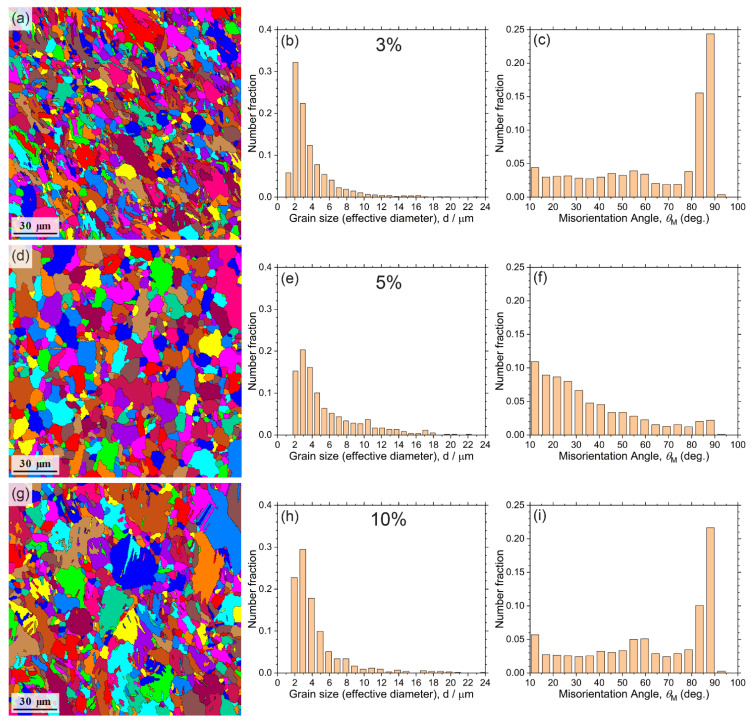
The grain structure of the alloy Mg-1Zn-0.2Ca alloy (**a**,**d**,**g**) and corresponding distributions of the effective grain size (**b**,**e**,**h**) and angles of misorientation between grains (**c**,**f**,**i**) after testing at 200 °C and $\dot{\varepsilon }$ = 5 × 10^−3^ s^−1^ to 3% (**a**–**c**), 5% (**d**–**f**), and 10% (**g**–**i**) strain.

**Figure 11:**
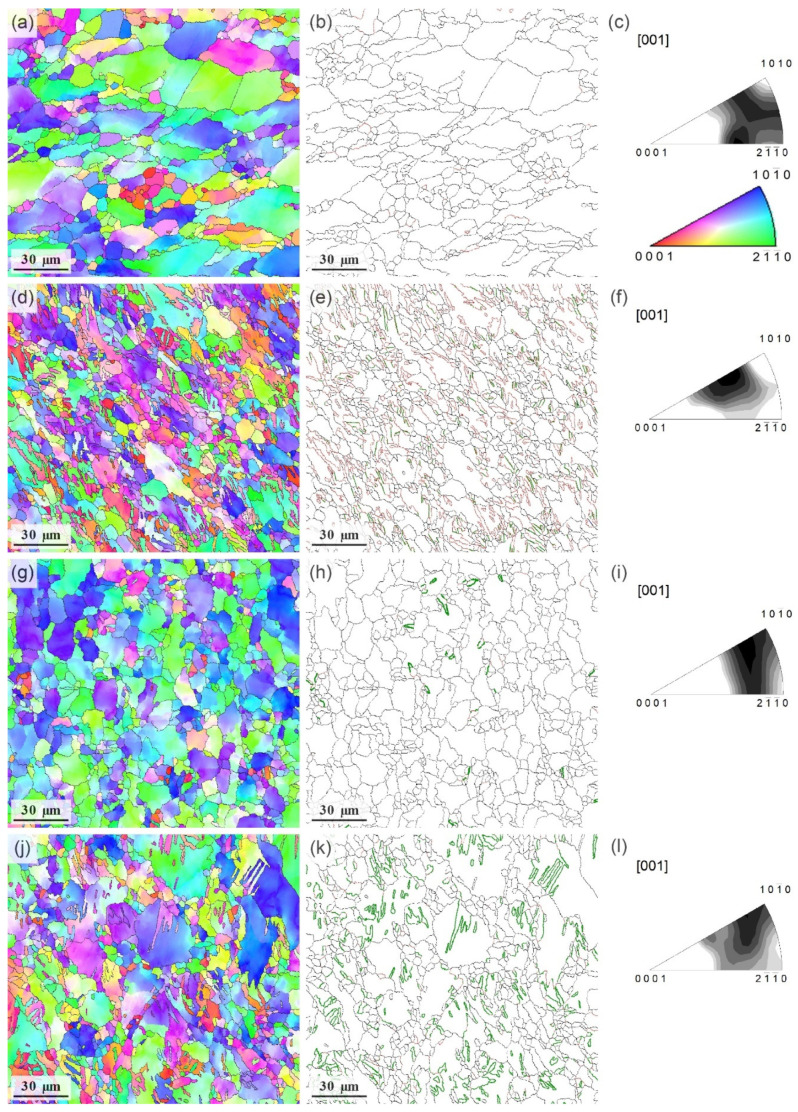
Results of the EBSD analysis of the deformation microstructure for the Mg-1Zn-0.2Ca alloy tested in tension at 200 °C to different strains (all maps have been obtained for the planes normal to the longitudinal axis of the specimens): (**a**–**c**) initial structure ε = 0, (**d**–**f**) ε = 3%, (**g**–**i**) ε = 5%, and (**j**–**l**) ε = 10%; (**a**,**d**,**g**,**j**) EBSD maps are coded in IPF colours shown in (**c**); (**b**,**e**,**h**,**k**) show the high-angle grain boundary maps with the highlighted (by the red colour) ranged between 85 and 95°, the tension twins are highlighted by green; (**c**,**f**,**i**,**l**) IPF pole figures indicating the texture evolution with straining.

The prime results of the investigation of the microstructure for the specimens tested at 200 °C and $\dot{\varepsilon }$ = 5 × 10^−3^ s^−1^ are shown in Figure 10 and Figure 11 At the strain stop of 3% ($<\varepsilon_{cr}^{AE}$), the microstructure consisted of strongly deformed grains, Figure 10a. Compared to the initial state, Figure 5b and Figure 11a–c, one can notice a greatly increased fraction of fine grains, Figure 10b and Figure 11d–f. Traces of DRX were clearly visible in the EBSD maps as well as in the inverse pole figures (IPF), Figure 11c,f,i,l. Dislocation-free grain nuclei formed firstly at a favourable boundary, which then led to the formation of necklace-type small recrystallised grains along the original grain boundaries of deformation-induced twins, which is known as a common signature of discrete dynamic recrystallisation [78] (see also comprehensive reviews by Humphreys et al. [57] and Sakai et al. [79]). Not surprisingly, the fine grains also nucleated at deformation twin boundaries, as is evidenced indirectly by the appearance of a strong high-angle component in the distribution of angles of misorientation, corresponding to that of extension twins in Mg (c.f., Figure 5c and Figure 10c). The role of deformation twinning in the formation of fine recrystallised grains is visible in Figure 11e in a more direct way. Fine-grain regions formed in the alloy during DRX did not show signs of twinning activities. As deformation proceeded to 5% ($\varepsilon_{cr}^{AE}$), c.f., Figure 10d and Figure 11g–h, notable grain coarsening and rounding occurred, giving rise to the increasing fraction of large grains, Figure 10e. The high-angle component in the distribution of misorientation angles decreased drastically, Figure 10f. As a result of these structural transformations, the material restored its ability to deform plastically. Indeed, as the strain increased to 10% ($>\varepsilon_{cr}^{AE}$), the grain size distribution remained almost unchanged (c.f., Figure 10e,h), but twin-related high angle boundaries reappeared in the deformation microstructure again, Figure 10i and, most notably, Figure 11k.

Similar changes in microstructure were observed for other testing schedules listed in Table 2, and, therefore, the described results are considered common and typical.

**Figure 12:**
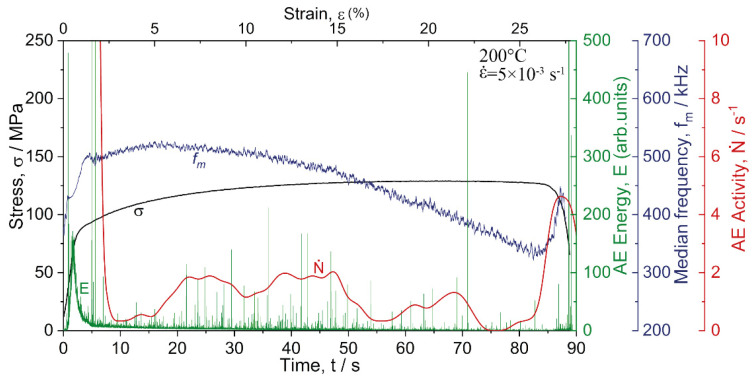
The acoustic emission diagram for the Mg-1Zn-0.2Ca alloy was tested at 200 °C (c.f., Figure 7f) with the activity (number of signals per second shown by a red line) of the high-amplitude twinning-related AE bursts superimposed.

As has been discussed above, the strain $\varepsilon_{cr}^{AE}$ is likely indicative of the ending of the DRX process when the nucleation of fine grains gives way to grain coarsening, thus regaining the capacity to accommodate plastic strains further. While there is no twinning activity in the small grains, the twins, which tend to nucleate easier at larger grains [80], the twins are supposed to reactivate in the grown grains. This is exactly what is seen in Figure 10g (implicitly in the distribution of the angles of misorientation) and Figure 11 (where extension twins are highlighted in the grain boundary EBSD maps). The careful examination of Figure 7f shows that after 18 s corresponding approximately to $\varepsilon_{cr}^{AE}$, the rapidly increasing AE activity was observed due to high amplitude AE bursts, which are attributed to twinning [81,82]. This is illustrated in Figure 12, where the activity of high-amplitude signals is plotted (red line) together with *E* and $f_{m}$ as a function of strain. It can be seen that this activity was maximum in the first 8 s of loading (up to 2% total strain). Then it decreased sharply, indicating exhaustion of the twinning mechanism. Between 8 and 18 s, the high amplitude transients ceased to appear. The AE median frequency increased, implying that the dislocation slip was a prevailing deformation mode at that time interval. Note that Barnett et al. [70] observed that twinning, which dominated the plastic flow during compression of AZ31 at lower temperatures, gave way to slip governing the plastic flow when the temperature increased. However, after 18 s, the activity of burst signals increased again, which is indicative of the recurrence of the deformation twinning in the recrystallised grains.

## 5. Conclusions

Motivated by the need for a better understanding of the deformation mechanisms underlying the mechanical behaviour of wrought Mg alloys in a wide range of temperatures and strain rates, we investigated the mechanical response of the low-alloyed fine-grain biomedical magnesium alloy Mg-1Zn-0.2Ca (in wt.%) fabricated via multiaxial isothermal forging and tested in tension with the concomitant measurements of the wideband acoustic emission signal. The following conclusions are drawn.

1. The common trends towards decreasing strength and increasing ductility were observed with the increasing test temperature; the steepest change occurred at 250 °C.
2. The advanced broadband AE technique was shown (for the first time to the best of our knowledge) to be well suited for reflecting the changes in the deformation behaviour associated with the dynamic recovery and recrystallisation occurring at elevated temperatures.
3. The evolution of the AE spectral density with plastic strain at elevated temperatures exhibited a specific behaviour of the median frequency, which peaked at a certain strain where dynamic recovery and recrystallisation occurred. This characteristic, strongly non-monotonic behaviour of the AE spectrum, which contrasts to the steady strain dependence at low temperatures, serves as an indicator of the evolution of dynamic recrystallisation towards completeness. In the investigated range of strain rates, the effect of dynamic recrystallisation was observed at 150 °C and higher.
4. The activity of mechanical twinning decreased with increasing temperature giving priority to dislocation slip. However, as recrystallisation proceeded and freshly nucleated grains grew, and the contribution of the twinning mode to the plastic flow regained and was visible even at elevated temperatures.
5. The proposed methodology based on the modern AE method can be efficiently adapted and applied in both laboratory research and in industrial settings to find the optimised manufacturing conditions for nominally hard to deform magnesium alloys.

## Figures and Tables

**Table 1:** Mechanical properties and acoustic emission parameters.

T, °C	$\dot{\varepsilon }$, s^−1^	$\sigma_{0.2},\;\mathrm{MPa}$	$\sigma_{UTS},\;\mathrm{MPa}$	$\varepsilon_{f},\;\%$	$\theta_{3–10},\;\mathrm{MPa}$	$\sigma_{0}^{AE},\;\mathrm{MPa}$	$\sigma_{\max}^{AE},\;\mathrm{MPa}$	$\varepsilon_{cr}^{AE},\;\%$
20	5 × 10^−4^	74 ± 2.5	193 ± 3	10.5 ± 0.5	734 ± 24	60 ± 4	93 ± 4	$>\varepsilon_{f}$
	5 × 10^−3^	77 ± 3	200 ± 3	12.5 ± 1	793 ± 29	23 ± 1	82 ± 3	$>\varepsilon_{f}$
	2 × 10^−2^	77 ± 3	210 ± 3	14 ± 1	835 ± 32	38 ± 2	74 ± 3	$>\varepsilon_{f}$
100	2 × 10^−2^	76	167	16	563	38	78	$>\varepsilon_{f}$
150	5 × 10^−4^	100 ± 4	141 ± 3	28 ± 2	222 ± 9	45 ± 2	82 ± 3	4.8 ± 0.2
	5 × 10^−3^	120 ± 5	162 ± 5	20 ± 2	224 ± 11	45 ± 2	73 ± 3	7 ± 0.3
	2 × 10^−2^	62 ± 2.5	148 ± 3	13 ± 1	490 ± 18	30 ± 2	58 ± 2	$>\varepsilon_{f}$
200	5 × 10^−4^	75 ± 3	108 ± 3	22 ± 2	178 ± 9	26 ± 3	72 ± 3	2.5 ± 0.1
	5 × 10^−3^	105 ± 4	140 ± 5	36 ± 5	182 ± 9	28 ± 3	68 ± 3	5.0 ± 0.2
	2 × 10^−2^	102 ± 4	135 ± 6	15 ± 1	237 ± 11	63 ± 4	80 ± 3	12 ± 0.4
250	5 × 10^−4^	56 ± 2	80 ± 3	96 ± 12	−58 ± 8	-	-	-
	5 × 10^−3^	65 ± 3	96 ± 3	30 ± 2	58 ± 7	35 ± 2	64 ± 3	1.7 ± 0.1
	2 × 10^−2^	70 ± 3	115 ± 5	25 ± 2	165 ± 10	23 ± 2	58 ± 3	2.8 ± 0.1
300	5 × 10^−4^	22 ± 2	30 ± 3	120 ± 30	−39 ± 20	-	-	-
	5 × 10^−3^	44 ± 3	57 ± 4	180 ± 35	−16 ± 8	-	-	-
	2 × 10^−2^	51 ± 3	63 ± 4	110 ± 20	19 ± 8	-	-	-

**Table 2:** Values of strain attained in the tests interrupted according to $\varepsilon_{cr}^{AE}$.

T, °C	Strain Rate, $\dot{\varepsilon }$, s^−1^	Strain (%)		
		$<\varepsilon_{cr}^{AE}$	$\varepsilon_{cr}^{AE}$	$>\varepsilon_{cr}^{AE}$
150	5 × 10^−3^	4.0	7.0	15
200	5 × 10^−3^	3.0	5.0	10
250	2 × 10^−4^	1.5	2.8	8.0

## Data Availability

Data are available from the corresponding author upon reasonable request.
